# The Many Faces of Huntington’s Chorea Treatment: The Impact of Sudden Withdrawal of Tiapride after 40 Years of Use and a Systematic Review

**DOI:** 10.3390/jpm12040589

**Published:** 2022-04-06

**Authors:** Stephanie Feleus, Malu van Schaijk, Raymund A. C. Roos, Susanne T. de Bot

**Affiliations:** 1 Department of Neurology, Leiden University Medical Center, P.O. Box 9600, 2300 RC Leiden, The Netherlands; 2 Department of Epidemiology, Leiden University Medical Center, P.O. Box 9600, 2300 RC Leiden, The Netherlands; 3 Intellectual Disability Medicine, Department of General Practice, Erasmus Medical Center Rotterdam, P.O. Box 2040, 3000 CA Rotterdam, The Netherlands; 4 Advisium, ‘s Heeren Loo, P.O. Box 647, 3800 AP Amersfoort, The Netherlands

**Keywords:** Huntington’s Disease, patients, tiapride hydrochloride, tiapridal, antipsychotics, neuroleptic, pharmacology, therapeutic use, chorea, movement disorders

## Abstract

Huntington’s Disease (HD) is a rare, neurodegenerative disorder characterized by chorea, cognitive decline, and behavioral changes. Despite wide clinical use since the mid-1980s, tiapride was recently withdrawn from the Dutch market without rationale. Although alternatives are available, many patients experienced dysregulation after this unwanted change. We provide insight into the impact of sudden tiapride withdrawal by reviewing medical records of HD patients who were using tiapride at the time of withdrawal. In addition, we performed a systematic search in five databases on tiapride efficacy and its safety profile in HD. Original research and expert opinions were included. In our patient group on tiapride, 50% required tiapride import from abroad. Regarding the review, 12 articles on original datasets and three expert opinions were included. The majority of studies showed an improvement in chorea while patients were on tiapride. Due to limited sample sizes, not all studies performed statistical tests on their results. Fifty percent of clinical experts prefer tiapride as initial chorea monotherapy, especially when comorbid behavioral symptoms are present. Side effects are often rare and mild. No safety concerns were reported. In conclusion, tiapride is almost irreplaceable for some patients and is an effective and safe chorea treatment in HD.

## 1. Introduction

Huntington’s Disease (HD) is an autosomal, dominantly inherited neurodegenerative disorder caused by an expansion of the CAG repeat in the huntingtin (HTT) gene [1,2]. The disease generally manifests during mid-life and is clinically characterized by involuntary movements (chorea), psychiatric and behavioral symptoms, and cognitive decline [3,4,5]. To this date, no cure or disease-modifying therapy exists [6,7]. However, a wide variety of pharmacological and non-pharmacological therapies are used to improve quality of life [7,8,9,10,11,12].

Traditionally, dopamine-blocking agents have been used to treat chorea [13,14]. Haloperidol, developed in the mid-20th century, was one of the first treatments [15,16,17]. In the mid-1980s, tiapride became available [18,19]. Tiapride was originally developed to counteract abnormal involuntary movements in various disorders [20,21,22]. Tiapride is marketed under various brand names, such as tiapridal, tiaprida, hipokin, sereprile, and tiaprid, and is highly affordable at approximately €0.55/day for 200 mg [23,24]. As an antipsychotic and benzamide derivate, tiapride acts by selectively blocking dopamine D_2_- and D_3_-receptors [25,26]. Blocking these dopamine receptors improves the regulation of behavioral, sleep, and motor function disturbances [26,27]. Therefore, tiapride is appealing to prescribe in clinically heterogeneous movement disorders such as HD and has been widely prescribed by European HD experts over the last 40 years [28,29,30].

In March 2020, Sanofi S.A. withdraw tiapride from the Dutch market abruptly [31]. This was the only company that supplied this medicine, which was covered by health insurance, in the Netherlands [31,32]. No major safety concerns were reported, whereas many HD patients reported benefits from tiapride [28,33]. The unexpected and sudden shortage of tiapride caused much distress among patients and their caregivers, as illustrated by three representative cases below [34]. Pharmacological alternatives, which had to be introduced quickly, were not always suitable and led to disease aggravation or (severe) side effects in several patients. Therefore, we reviewed the records of all our HD patients who were using tiapride at the time of withdrawal and made a total overview of the subsequent adjustments and the success rate of substitute medicines given. In addition, a systematic literature search was performed to review the effectiveness and safety profile of tiapride in HD. Side effects and potential adverse drug effects are summarized.

## 2. Case Descriptions

A 60-year-old man was clinically and genetically diagnosed with HD in 2013. His chorea was well controlled with tiapride 100 mg three times a day without any side effects. After market withdrawal, tiapride was tapered down to 50 mg per week and tetrabenazine was started. After three days of tetrabenazine 12.5 mg, the patient contacted the hospital because of severe nausea and dizziness, requiring domperidone. Tetrabenazine was stopped and tiapride reintroduced in increasing doses up to 300 mg per day again and imported at the patient’s own expense. No other explanation for the nausea, such as food poisoning or infection, was found. The nausea disappeared within a few days and after two weeks the patient felt like his normal self again.

A 66-year-old female HD patient with coexisting sleep problems and urge incontinence had been taking tiapride since 2016. Her chorea was well controlled with 150 mg daily without side effects. After market withdrawal, tiapride was tapered down and her chorea increased enormously. The patient’s UHDRS-TMS (Unified Huntington’s Disease Rating Scale—Total Motor Score, with a range from zero to 124 [35]) increased in one year from 37 to 82. This was mostly due to chorea and dystonia, with a 24- and 12-point increase, respectively. Initiation of quetiapine 25 mg daily did not affect chorea but led to nausea and frequent vomiting as side effects. As a second alternative tetrabenazine was started up to three times a day 25 mg. Her chorea improved, but the nausea persisted, and her urge incontinence worsened. Switching to another urinary spasmolytic decreased the urinary frequency. Nausea was partly reduced by dietary measures. A year after tetrabenazine initiation, the patient’s UHDRS-TMS had stabilized at 45 points. In consultation with the patient and her family, it was decided to continue tetrabenazine 25 mg three times daily, although side effects (mostly nausea) persisted.

A 60-year-old man with clinically and genetically diagnosed HD had an extensive cardiovascular medical history and diabetes mellitus type 2 with renal failure that left him dependent on hemodialysis. For HD symptoms, he used mirtazapine 45 mg and tiapride 100 mg twice daily. Since tiapride was no longer available, tetrabenazine was started at 25 mg three times a day. Although no side effects occurred, tetrabenazine was not effective enough and his increased choreatic movements hindered hemodialysis substantially. Hemodialysis was aborted on several occasions and additional haloperidol had to be administered intravenously. The patient then switched back to low doses of tiapride, with good antichoreatic control and no further need for haloperidol.

## 3. Materials and Methods

Leiden University Medical Center is a large HD expertise center in the Netherlands with more than 500 patient visits per year. Medical records of all Huntington’s Disease patients who were using tiapride at the time of withdrawal, in March 2020, were reviewed. To include all patients, all medical records were searched for tiapride use between 1 January and 31 December 2020. If no hospital tiapride prescription was present during the period of interest, the national healthcare network (Dutch: Landelijk Schakelpunt) for medication prescriptions was consulted. We analyzed what happened after tiapride market withdrawal for each patient. Possible options included tapering down of tiapride, starting a pharmacological alternative within 3 months, and/or tiapride import from abroad. The latter required patients to pay in advance (±€200/year), without certainty of insurance reimbursement.

The databases PubMed, Web of Science, PsychInfo, Embase, and the Cochrane Library were all searched from their respective inception up to 17 November 2021 using a systematic search. This search focused on tiapride and Huntington’s Disease. Synonyms tiapridal and tiaprid were included. Included languages were English, Dutch, German, and French. Original studies, in which effects (either positive or negative) of tiapride in genetically proven (CAG-repeat length ≥36) HD patients were described, and articles (including expert opinions) that described pharmacological alternatives to tiapride in HD were included. Case reports, book chapters, research protocols, and Huntington’s Disease-like syndromes were excluded. The complete search strategy can be found in the  Supplementary Materials. All records were screened independently by two authors, Stephanie Feleus and Malu van Schaijk, for English and Dutch articles, or Stephanie Feleus and Raymund A.C. Roos for German and French articles. In case of disagreement on whether to include a record in the next screening phase or not, records were given the benefit of the doubt and stayed in screening. Selected results were screened in full-text and references of included articles were cross-checked for additional records. If a relevant new record was identified based on title, it would enter the phase of title-abstract screening in the selection process.

The tiapride package leaflet and the World Health Organization (WHO) VigiAccess database were consulted for definite and potential side effects of tiapride on 17 November 2021, respectively [33,36]. VigiAccess is a web-based platform that searches VigiBase, the WHO global database of individual case safety reports. VigiAccess data come from a variety of sources, including but not limited to patients, medical practitioners, pharmaceutical companies, and worldwide national regulatory authorities [37]. Drug relatability is variably assessed by data-providing countries, which means that causality cannot be inferred. Since only records that are documented are included and the total number of patients on tiapride is unknown, incidence cannot be assessed, either. To provide an overview of rarely reported potential side effects, adverse drug reactions that were reported 25 times or more (cut-off arbitrarily chosen) in VigiAccess and are not otherwise listed in the patient leaflet are described in this review.

## 4. Results

### 4.1. Evaluation of Medical Records

**Figure 1:**
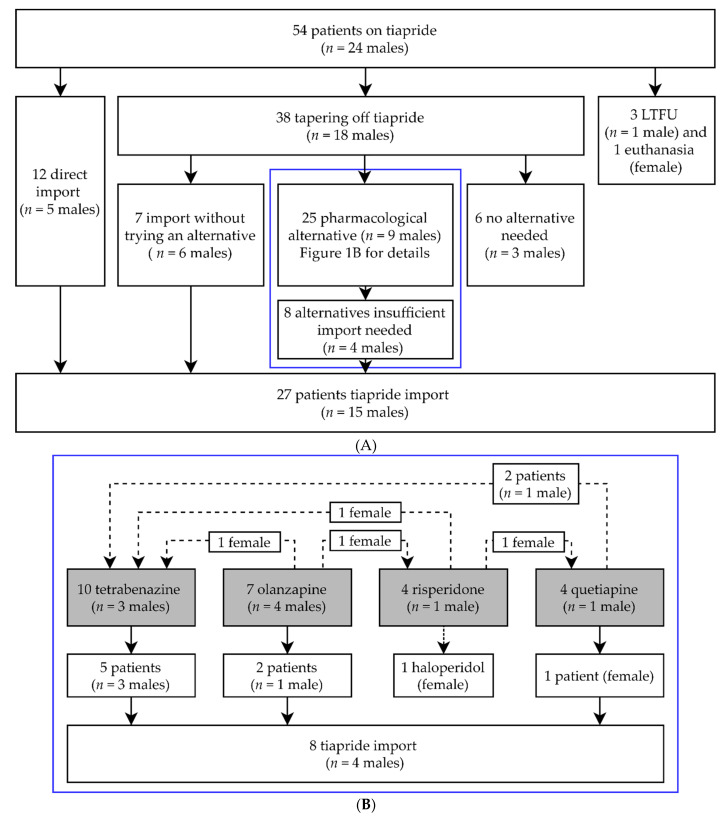
(**A**) **General overview of clinical follow-up and tiapride import.** In the blue box are those patients that were prescribed a pharmacological alternative. (**B**) describes these pharmacological alternatives in more detail; *n* = number of; LTFU = Lost to follow-up. (**B**) **Enlargement of blue box** (**A**). **Details of pharmacological alternatives used.** Gray boxes are the original pharmacological alternatives prescribed. Dotted lines are patients who switched between pharmacological alternatives; *n* = number of.

Of all our HD patients, 54 patients (mean age 58.0 years (range 23–86 years), *n* = 24 males) used tiapride at the time of withdrawal in March 2020. Without first tapering off and trying an alternative, 12 patients (mean age 54.6 years) directly chose tiapride import from abroad at their own expense. For the majority of patients, tiapride was first tapered off (*n* = 38, mean age 59.6 years). Four patients were either lost to follow-up or passed away (mean age 59.8 years). Details are shown in Figure 1A. No pharmacological alternative was needed for 6 patients (mean age 59.7 years). Most of them were using a low dose (≤100 mg daily), which could explain why no alternative was needed. During tapering off or directly after, seven patients (mean age 56.0 years) required immediate tiapride import (Figure 1A). Twenty-five patients (mean age 59.6 years) were prescribed alternative medications. The alternatives that were used most often were tetrabenazine and olanzapine. Less regularly chosen were quetiapine and risperidone. Several patients switched between alternatives. From those prescribed an alternative, a minority of patients (40%) continued with their first alternative (data not shown). Ultimately, 17 of the 25 patients (68%) stayed on their final pharmacological alternative. A graphical overview is shown in Figure 1B. Finally, for 27 patients (50% of the total group) tiapride needed to be imported. The average age of these 27 patients (*n* = 15 males) was 55.6 years. 

### 4.2. Systematic Literature Review on Tiapride in Huntington’s Disease

**Figure 2:**
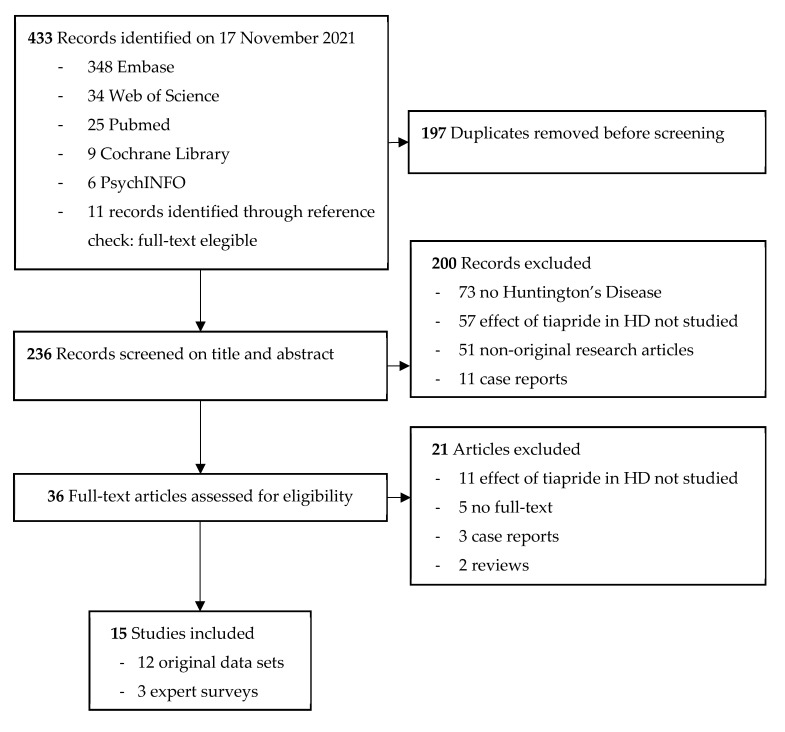
Flow diagram of selected studies.

The literature search performed yielded 433 results. After removing duplicates, 236 studies were screened for title and abstract, and 36 studies remained for full-text screening. We were unable to retrieve the full text of five articles [38,39,40,41,42]. When screening the full texts, another 16 articles did not meet the inclusion criteria. Cohen’s kappa (κ) for inter-rater variability between SF, MS, and RACR combined is 0.72, which indicates a substantial interrater agreement. Ultimately, 15 studies were included, of which 12 were original articles and three were expert surveys on prescription preferences. An overview of the selection process with reasons for exclusion is shown in Figure 2.

Only a small number of studies have explored the treatment potential of tiapride in HD. A detailed overview of these studies is given in Table 1. The majority, nine out of 12, were experimental studies with a time frame ranging from several weeks to one year [18,19,43,44,45,46,47,48,49]. Dosing of tiapride varied greatly between studies, ranging from 300 to 3000 mg/day [18,19,43,44,45,47,48,49]. Many studies used an effect-guided titration scheme for tiapride with an equilibrium between 300 and 900 mg/day [18,43,44,45,47,48]. The largest, although still small with *n* = 23, experimental, placebo-controlled trial was performed by Deroover et al. [19]. In patients with early to moderate HD, they found a significant chorea improvement (*p* < 0.05) with tiapride 3000 mg/day for 6 weeks. These results are strengthened by several other studies, which already showed a positive effect on chorea control with lower dosages ranging from 300 to 1200 mg/day [18,43,45,46,47,48]. The second-largest experimental study by Roos et al. was also a placebo-controlled, double-blind, cross-over trial with 22 HD patients on tiapride 300 mg/day for two weeks [49]. With a relatively short follow-up period, merely a trend towards significance (*p* = 0.10) was found. On the contrary, Girotti et al., who prescribed tiapride 600–800 mg/day in 12 patients for 4 weeks, found no significant chorea improvement relative to pimozide and haloperidol, which gave more side effects [44]. One of the more recent observational studies by Cichecki et al. performed an uncorrected analysis on 20 HD patients with varying although stable tiapride dosages [50]. No significant difference in UHDRS-TMS between baseline and one-year follow-up was found. Only a minority of studies reported the number of males/females at baseline [19,44,47,49,51]. Two studies analyzed potential sex differences in tiapride response. Petit et al. was the only study that described that females might have a reduced tiapride response [47]. Roos et al. could not reproduce these results [49]. In summary, most studies, seven out of 11 that focused on abnormal movements, concluded that tiapride has a positive effect on chorea. None of the studies reported a significant worsening of chorea with tiapride.

Although most research has been focused on chorea control, two observational studies described the effect of tiapride on body weight [51,52]. Konvalinkova et al. found no significant weight change, neither weight gain nor weight loss, in 49 patients on tiapride compared to 260 controls [52]. Desamericq et al. reported less weight gain (*p* < 0.05) with tiapride compared to known weight increasers olanzapine and risperidone in a similar-sized population [51]. Based on these two studies, tiapride appears not to affect body weight [51,52]. As a comparative study, Desamericq et al. commented on functionality changes in different drug groups [51]. Functionality, measured by the Unified Huntington’s Disease Rating Scale—Total Functional Capacity (UHDRS-TFC), describes one’s capacity to live independently and perform activities of daily living [35]. Functional deterioration was more pronounced (*p* < 0.05) in patients on tiapride than in those on dibenzodiazepines (mostly olanzapine). However, tiapride was given to more diseased HD patients at baseline, so the unadjusted significant decline in UHDRS-TFC tends to be confounded by this. Only one included study was designed to test the efficacy of tiapride on depressive symptoms, anxiety, and irritability in HD [19]. However, at baseline most patients did not experience these psychiatric symptoms. Therefore, the power to deduct any conclusion hereof is too low.

### 4.3. Safety and Side-Effect Profile of Tiapride

Treatment with tiapride is generally well tolerated, particularly in HD patients (Table 1). Side effects are often rare and mild. After four decades of tiapride use in clinical practice, the most reported (in 1–10% of patients) side effects are somnolence, apathy, agitation, and during the beginning of treatment, rigidity, hypokinesia, and hypersalivation [19,24,44,46,47,49]. Less commonly (0.1–1%) reported side effects are akathisia and symptoms that are a consequence of elevated prolactin levels, such as gynecomastia, galactorrhea, and weight increase [24,46,47]. For detailed descriptions of rare side effects, we refer to the package leaflet [36]. In VigiAccess, 1897 reports of suspected potential side effects were present since tiapride became available. This is a relatively small number of reports considering that tiapride has been available for over four decades and is also used in psychiatric patients. Only those symptoms that were reported 25 times or more (arbitrarily chosen) and that are not otherwise mentioned in the patient leaflet are listed here: confusional state (*n* = 94), hyponatremia (*n* = 36), urinary retention (*n* = 34), loss of consciousness (*n* = 33), bradycardia (*n* = 27), thrombocytopenia (*n* = 26), increased serum creatine phosphokinase (*n* = 26), aspiration pneumonia (*n* = 26), and rash (*n* = 25). Simultaneous use of tiapride with antidepressants, benzodiazepines, analgesics, opiates, or alcohol might increase its sedative side effects [24]. Simultaneous use of tiapride with substances that might prolong QT-interval, such as class IA and III antiarrhythmic agents, domperidone, haloperidol, and pimozide, should be avoided [36].

### 4.4. Expert Opinions

Three expert surveys were included in this review. Two studies focused on pharmacological chorea control [28,53]. One article summarized preferred treatment options for irritability in HD [29]. A summary of the included expert opinions can be found in Table 2. The survey with the most respondents (*n* = 200) was conducted among USA neurologists and describes pharmacological treatment options for HD [53]. At the time of publication, solely tetrabenazine was FDA-approved for HD chorea control. Tiapride was and is still not available in North America. However, 54% of respondents perceived tetrabenazine as having minimal or no effectiveness in suppressing HD chorea. Antipsychotics (26%) and amantadine (9.3%) were reported as off-label alternatives. The most commonly prescribed antipsychotics were risperidone (6.9%), quetiapine (6.7%), and haloperidol (5.8%). Burgunder et al. conducted a multinational survey among North American, European and Australian physicians [28]. Regional differences were visible since respondents from North America and Australia equally favored off-label antipsychotics (58%) or tetrabenazine (56%), while European respondents evidently preferred antipsychotics (87%) and specifically tiapride (50%) as initial monotherapy. Tetrabenazine was considered an alternative monotherapy by 67% of European respondents. All experts preferred antipsychotic monotherapy when comorbid behavioral symptoms were present. A combination of tetrabenazine and an antipsychotic was prescribed when symptoms were inadequately controlled by monotherapy. Efficacy and side-effect profiles were considered similar for antipsychotics and tetrabenazine, except that depression is more prevalent in the latter. 

One expert opinion focused on the pharmacological treatment of irritability [29]. Worldwide respondents preferred antipsychotics (77%) for severe aggression. In case of milder irritability symptoms, SSRIs (57%), antipsychotics (31%), and mirtazapine (28%) were endorsed. Olanzapine, risperidone, and, to a lesser extent, tiapride are specific drugs from the antipsychotic class that were favored. None of the studies reported sex differences in tiapride treatment response [28,29,53].

## 5. Discussion

To our knowledge, this is the first review on the efficacy and safety profile of tiapride in HD. A general beneficial trend is seen for tiapride as chorea treatment in HD. Although we were unable to retrieve the full text of five articles, we do not expect our conclusion to change if those articles had been available. One study described potential decreased tiapride efficacy in females [47]. However, treatment duration differed between sexes. Females who did not respond to tiapride treatment were all treated for three weeks or less. In comparison, the treatment period for males was often several months or more. None of the other included studies reported any sex differences in tiapride response. Therefore, it seems unlikely that tiapride affects men and women differently. This is supported by our own patients’ data. The small number of studies included in our review were inconclusive about the effect of tiapride on the weight of HD patients, but research in other patient populations has demonstrated that tiapride sporadically may lead to weight gain [21]. Weight increase is limited to some kilograms during the beginning of treatment. Major weight gain is rarely reported [54] and is likely a consequence of increased prolactin levels and increased appetite [55]. No recommendations for specific disease stages can be made since not all studies described disease severity in their study population (data not shown) and all subgroups were small. Two studies have explored this. Deroover et al. described an increased efficacy in patients in disease stage II, compared to those in stage I of the Shoulson and Fahn HD disease stage classification [56], while Roos et al. were not able to distinguish patients in whom chorea improved based on severity or duration of illness [49]. Larger studies with patient and disease characteristics including disease severity are needed to investigate who benefits most from tiapride and at what dose.

This review showed that side effects of tiapride are often rare and mild. Somnolence is one of them [36]. Prescribing tiapride in the evening could not only treat restlessness during the night but may also benefit pre-existing sleeping problems that are common in HD [57,58]. For tetrabenazine, the most often used pharmacological alternative, caution is advised in those patients who have experienced or currently experience depressive symptoms [59,60]. In addition, nausea can be a limiting side effect of tetrabenazine [59,61]. Tiapride has a more favorable profile considering these points [36]. Antipsychotics, especially those belonging to the first generation, could induce tardive dyskinesia with prolonged use [62]. This iatrogenic syndrome is characterized by involuntary movements most pronounced in the face and oromandibular region. To this date, only a very small number (*n* = 22) of potential cases of tardive dyskinesia related to tiapride have been described in VigiAccess [33]. These cases did not meet our threshold of being reported 25 times or more. In none of the cases causality was confirmed. The risk of developing tardive dyskinesia is related to a drug’s potency to block the dopaminergic D_2_-receptors [63]. Even at a higher dosage of 600 mg/day, tiapride does not exceed an 80% D_2_-receptor occupancy [64]. This could very well be a protective factor against developing tardive dyskinesia. Moreover, in some countries tiapride is registered as a substitute antipsychotic when tardive dyskinesia is present.

Limitations of our review are mostly based on the nature of included studies. Most studies were conducted in the 1970s and 1980s, according to the standards of that time [18,19,43,44,45,46,47,48,49]. Relatively small sample sizes (*n* < 25) were common [18,19,43,44,45,46,47,48,49,50]. Nearly half of the included articles with original data did not perform statistical tests but reported clinically observed changes instead [18,45,46,47,48]. Different outcome measures were used (Table 1). A formal meta-analysis was therefore not possible. Only two out of 12 therapeutical studies used the UHDRS-TMS as an outcome measure [50,51]. Developed in 1996, this scale is the gold standard nowadays for systematically assessing chorea and other physical characteristics of HD [35]. The majority (nine out of 12) of the included therapeutic studies are dated before 1996 and used various outcome measures instead. These include clinical observation by the researcher, patient and/or caregiver, subjective measures on Likert scales, and qualitative and quantitative scores, such as the Abnormal Involuntary Movement Scale (AIMS) [65]. Studies included in our review also varied substantially in treatment periods and dosages of tiapride. Treatment periods ranged from one week up to eight years (Table 1). Dosages of tiapride ranged from 300 mg/day to 900 mg/day with an outlier of 3000 mg/day [18,19,43,44,45,47,48,49]. Unfortunately, several studies did not even report the administered dosage or treatment period [46,48,50,51,52]. Deducting the optimum dosage is therefore difficult. The dosages most often reported were between 300 and 900 mg/day depending on chorea severity and treatment response. The great diversity in study design further complicates the comparability of studies.

Tiapride is not available in the USA for reasons unknown and unavailable to us. As a consequence, our results show a strong regional preference determined by availability in Europe. Prescribing preferences in countries outside Europe, USA, and Australia are concealed to us. Since European clinicians have more experience with tiapride, it inclines them to prescribe tiapride sooner in the next patient. To include as many records as possible, we enriched our search by including French and German language articles.

We do not know the underlying reasons to abruptly withdraw tiapride from the Dutch market. No safety issues have been reported. Business decisions for a cheap, infrequently used drug could have played a role for Sanofi S.A., a large, international pharmaceutical company. Its decision to withdraw tiapride from one national market might jeopardize other countries as well. The sudden lack of tiapride required health care professionals to swiftly provide pharmaceutical alternatives in a complex patient population while being in the middle of the COVID-19 pandemic lockdown. Alternatives were not suitable for more than half of our patients, and a trial and error of medication was often needed before finding an effective alternative. Reasons based on certain patient characteristics to choose tiapride in the first place could have enlarged this percentage. Several reviews have been conducted in the last years, summarizing possible treatment options for chorea in HD [8,30,66]. Alongside (deu)tetrabenazine, these include quetiapine, olanzapine, risperidone, sulpiride, clozapine, aripiprazole, haloperidol, amantadine, lorazepam, and clonazepam. Aside from varying degrees of expected benefit, each drug has its advantages and disadvantages. Based on the information currently available on tiapride in HD, a large RCT to investigate the true effect of tiapride would be beneficial. A French comparative study, called NEUROHD, is ongoing and will publish its results in the near future [67]. In this multi-center, randomized clinical trial with 180 HD patients, the effects of olanzapine, tetrabenazine, and tiapride on patients’ functionality are compared. Secondary endpoints include motor function, psychiatric and cognitive performance, metabolic parameters, drug tolerance, and cost aspects. However, this is not an approach that takes into account personalized medicine, where patient characteristics such as disease characteristics, pharmacogenetic profile, and comorbidities, could in fact dictate the most effective medicine on an individual basis. Further studies to disentangle these specific characteristics are needed.

## 6. Conclusions

The available studies on tiapride included in our study do not always meet current research standards. However, a general beneficial trend is seen for tiapride as an anti-choreatic treatment in HD. Interestingly, while tetrabenazine is the only approved anti-choreatic treatment in many countries, many experts in the HD field recommend using antipsychotics as initial monotherapy. Based on regional availability, specific drugs are recommended. In Europe, tiapride is favored by many patients and by half of the clinicians. Furthermore, tiapride is cheap and considered to be well tolerated. Over the past decades, no major safety concerns have arisen. Common side effects are mild and often temporary. In line with the International Therapeutic Guidelines [30], we recommend that clinicians consider tiapride treatment for HD chorea, in particular when the patients have associated personality and/or behavioral or psychotic disorders. 

## Figures and Tables

**Table 1:** Studies on tiapride in Huntington’s Disease.

Reference	*n* HD Patients (*n* of Males)	Type of Research	HD Symptom	Tiapride Dosage	Time Frame/Follow-Up	Outcome	Side Effects of Tiapride
Chouza et al., 1982 [18]	2 (uk)	Experimental, double-blind, placebo-controlled	Chorea	Build-up scheme, max 900 mg/day	13 weeks	Subjective moderate–important improvement of chorea by patient, caregiver, and observer	Psychomotor excitation and suicidal thoughts at 900 mg/day in one patient
Cichecki et al., 2016 [50]	20 (uk)	Observational, no control group	Chorea	Varies, unknown. Each patient had a stable dosage for 52 weeks.	52 weeks	Non-significant difference in UHDRS-TMS at BL 36.9 ± 3.7 and at FU 40.3 ± 4 (*p* > 0.05)	None reported
Csanda et al., 1984 [43]	15 (uk)	Experimental	Chorea	600–900 mg/day	52 weeks	Significant improvement in AIMS score (BL ±11 and FU ±5.5, *p*-value not given)	None reported
Deroover et al., 1984 [19]	23 (11)	Experimental, cross-over randomized, double-blind, placebo-controlled	Chorea, Depression, Anxiety, Irritability	3000 mg/day	9 weeks, of which tiapride was administered twice for 3 weeks at a time	Chorea significantly improved on Likert 4-point scale (*p* < 0.05). Leeds psychomotor test for reaction time significantly faster with tiapride (*p* < 0.01). Insufficient data to draw conclusions on psychiatric symptoms	Mild sedation was common, some patients experienced extrapyramidal effects
Desamericq et al., 2014 [51]	347 patients of which 43 (26) on tiapride	Observational, tiapride compared to dibenzodiazepines (olanzapine), risperidone, and tetrabenazine	Chorea, Functionality (UHDRS-TFC), Weight loss	Varies, unknown	Mean 122 weeks, max 8 years	No significant difference on UHDRS-TMS. More decline in functionality compared to olanzapine (*p* < 0.05). Less weight gain on tiapride (*p* < 0.05) compared to olanzapine or risperidone	None reported
Girotti et al., 1984 [44]	18 (11), of which 12 (uk) received tiapride	Experimental, cross-over, tiapride compared to pimozide 12–16 mg/day and/or haloperidol 6–9 mg/day	Chorea	600–800 mg/day	13 weeks, of which 4 weeks tiapride	No significant improvement in chorea on the AIMS score with tiapride, while pimozide (*p* < 0.01) and haloperidol (*p* < 0.05) significantly improved the AIMS score.	Fewer side effects of tiapride compared to pimozide and haloperidol (both more somnolence, rigidity and dysarthria)
Grass et al., 1983 [45]	4 (uk)	Experimental, no blinding	Chorea	Build-up scheme, max 600 mg/day	2 weeks	Subjective good–very good improvement in chorea by observer. Supported by EMG measurements	None reported
Konvalinkova et al., 2018 [52]	49 (uk) on tiapride, 260 (uk) without tiapride	Observational, cross-sectional	Weight loss	Unknown	NA	No significant weight difference between those with tiapride and those without	None reported
Mathe et al., 1978 [46]	6 (uk)	Experimental, no blinding	Chorea	Varies, unknown	Varies, at least 1 week, details unknown	Notable improvement in chorea and in more severely diseased patients some improvement in functionality (e.g., writing)	Temporary somnolence, galactorrhea and/or blood pressure increase in single patients
Petit et al., 1979 [47]	22 (13)	Experimental, no blinding	Chorea	300–600 mg/day	Mean 31 weeks, max 26 months	Subjective moderate–good improvement on chorea	Weight increase in some patients, apathy, and/or somnolence
Quinn et al., 1985 [48]	7 (uk)	Experimental, placebo-controlled	Chorea	600–1200 mg/day	Unknown	Median abnormal movement count improved by 42%, chorea score by 6.5%, and functional score by 18.4%	None reported
Roos et al., 1982 [49]	22 (9)	Experiment, cross-over, double-blind	Chorea	300 mg/day	4 weeks, of which 2 weeks tiapride	No significant improvement in involuntary movement count	In minority of patients, mild drowsiness

Legend: AIMS = Abnormal Involuntary Movement Scale; BL = Baseline; EMG = Electromyography; FU = Follow-up; HD = Huntington’s Disease; mg = milligram; NA = not applicable; *n* = number of; *p = p*-value; UHDRS-TFC = Unified Huntington’s Disease Rating Scale—Total Functional Capacity; UHDRS-TMS = Unified Huntington’s Disease Rating Scale—Total Motor Score; uk = unknown.

**Table 2:** Summary of included expert surveys.

Reference	*n* Respondents	Practice Based in	HD Symptom	Initial Monotherapy without Comorbidities Present	Side Effects of Tiapride
Sung et al., 2018 [53]	200	100% USA	Chorea	Respondents preferred tetrabenazine (50%). Antipsychotics (26%) were considered off-label alternatives.	NA *
Burgunder et al., 2011 [28]	52	42% EU 54% USA/Canada 4% Australia	Chorea	Worldwide respondents preferred antipsychotics (58%) as a first choice, and tetrabenazine (56%) as an alternative monotherapy. Fifty percent of EU respondents preferred tiapride. * Risperidone (43%) and olanzapine (39%) were also preferred. ^×^	Sedation, Parkinsonism
Groves et al., 2011 [29]	55	47% EU 49% USA/Canada 4% Australia	Irritability	Respondents preferred an SSRI (57%) as first choice, and antipsychotics (31%), mirtazapine (28%), or anti-epileptic drugs (27%) as alternative monotherapy. Twenty-seven percent of EU respondents preferred tiapride. *	None reported

Legend: * Tiapride is not available in North America; ^×^ The total is more than 100% since each respondent could check more than one option.

## Data Availability

Due to privacy restrictions, patients’ medical records are not available.

## Supplementary Materials

The following are available online at https://www.mdpi.com/article/10.3390/jpm12040589/s1, S1: Search strategy.

## Abbreviations

AIMS = Abnormal Involuntary Movement Scale; BL = Baseline; CAG-repeat = Cytosine Adenine Guanine trinucleotide repeat; EU = Europe; EMG = Electromyography; FDA = United States (of America) Food and Drug Administration; FU = Follow-up; HD = Huntington’s Disease; HTT = Huntingtin; LTFU = Lost to follow-up; mg = milligram; *n* = number of; NA = Not applicable; *p* = *p*-value; RCT = Randomized Controlled Trial; UHDRS- TFC = Unified Huntington’s Disease Rating Scale—Total Functional Capacity; UHDRS-TMS = Unified Huntington’s Disease Rating Scale—Total Motor Score; uk = unknown; USA = United States of America; WHO = World Health Organization.
