# Supplementary material for: The Many Faces of Huntington’s Chorea Treatment: The Impact of Sudden Withdrawal of Tiapride after 40 Years of Use and a Systematic Review
Source: J Pers Med. 2022 Apr 6;12(4):589. doi: 10.3390/jpm12040589 (PMC9025785; doi:10.3390/jpm12040589)
Supplement: Supplementary file 1 [file jpm-12-00589-s001.zip › jpm-1632968-supplementary.pdf]

SUPPLEMENTARY MATERIAL
A. Search strategy

Inclusion criteria:
- Original research (not necessarily peer-reviewed). Recent poster presentations and conference abstracts allowed.
- Descriptions of (possible) effects, either positive or negative, of tiapride on Huntington's Disease symptoms. Surveys among Huntington's Disease experts and clinicians are allowed.
- Genetically proven Huntington's Disease. CAG repeat length ≥ 36.
- Human or animal model.
- Language: English, Dutch, German or French.
- No date of publication restrictions.

Exclusion criteria:
- Book chapters, research protocols.
- Case reports (*n* = 1).
- No Huntington's Disease, e.g. Huntington's Disease-like syndromes etc.

PubMed
Search conducted on 17 November 2021
("tiaprid"[All Fields] OR "tiapride hydrochloride"[MeSH Terms] OR ("tiapride"[All Fields] AND "hydrochloride"[All Fields]) OR "tiapride hydrochloride"[All Fields] OR "tiapridal"[All Fields] OR "tiapride"[All Fields] OR ("tiaprid"[All Fields] OR "tiapride hydrochloride"[MeSH Terms] OR ("tiapride"[All Fields] AND "hydrochloride"[All Fields]) OR "tiapride hydrochloride"[All Fields] OR "tiapridal"[All Fields] OR "tiapride"[All Fields]) OR ("tiaprid"[All Fields] OR "tiapride hydrochloride"[MeSH Terms] OR ("tiapride"[All Fields] AND "hydrochloride"[All Fields]) OR "tiapride hydrochloride"[All Fields] OR "tiapridal"[All Fields] OR "tiapride"[All Fields])) AND ("huntington"[All Fields] OR "huntington s"[All Fields] OR "huntingtons"[All Fields] OR ("huntington"[All Fields] OR "huntington s"[All Fields] OR "huntingtons"[All Fields]))

Web of Science
Search conducted on 17 November 2021
All databases: ((TS=(tiapride)) OR TS=(tiapridal) OR TS=(tiaprid)) AND (TS=(Huntington) OR TS=(Huntington's))

PsychINFO
Search conducted on 17 November 2021
TX ((tiapride OR tiapridal OR tiaprid)) AND TX ((Huntington OR Huntington's))

Embase
Search conducted on 17 November 2021
((tiapride or tiapridal or tiaprid) and (Huntington or Huntington's)).af.
All resources: University Leiden Ovid JournalsJournals@Ovid Full Text <November 17, 2021>University Leiden Ovid BooksEmbase <1974 to 2021 November 17>ERIC <1965 to November 2021>Ovid Emcare <1995 to 2021 Week 46>Inspec Archive - Science Abstracts <1898 to 1968>Ovid MEDLINE(R) ALL <1946 to November 17, 2021>

Cochrane Library
Search conducted on 17 November 2021

((tiapride or tiapridal or tiaprid) AND (Huntington OR Huntington’s)) in Title Abstract Keyword
